# Responses of the cutworm *Spodoptera litura* (Lepidoptera: Noctuidae) to two Bt corn hybrids expressing Cry1Ab

**DOI:** 10.1038/srep41577

**Published:** 2017-02-10

**Authors:** Shu Yinghua, Du Yan, Chen Jin, Wei Jiaxi, Wang Jianwu

**Affiliations:** 1 Key Laboratory of Agro-Environment in the Tropics, Ministry of Agriculture, South China Agricultural University, Guangzhou 510642, China; 2 Key Laboratory of Agroecology and Rural Environment of Guangdong Regular Higher Education Institutions, South China Agricultural University, Guangzhou 510642, China; 3 Department of Ecology, College of Natural Resources and Environment, South China Agricultural University, Guangzhou 510642, China; 4 Department of Crop Science and Technology, College of Agriculture, South China Agricultural University, Guangzhou 510642, China

## Abstract

To examine the responses of the secondary lepidopteran pest *Spodoptera litura* to two *Bacillus thuringiensis* (Bt) corn hybrids [5422Bt1 (Event Bt11), 5422CBCL (MON810)] expressing Cry1Ab, larval bioassays with Cry1Ab toxin, corn leaves or kernels and bagging on corn plants were conducted. The results showed that larvae displayed a similar performance when fed kernels, but not leaves of 5422Bt1, 5422CBCL and their near-isogenic non-Bt corn (5422). Significantly higher Cry1Ab amounts were detected in larvae fed leaves than kernels of both Bt hybrids, with different molecular weights of protein band in plants (72 and 90 kDa for 5422Bt1 and 5422CBCL, respectively), gut contents (65 kDa), feces (50 kDa), which indicated that larvae had lower ingestion, higher degradation and excretion of Cry1Ab when fed kernels not leaves of both Bt hybrids. Significantly higher levels of cadherin-like receptors and alkaline phosphatase transcripts were detected in larvae fed leaves than kernels of two Bt hybrids. Catalase, superoxide dismutase and glutathione-S-transferase activities in larvae fed 5422Bt1 leaves were significantly higher than that of 5422 treatments. Therefore, *S. litura* had low susceptibility to 5422Bt1 and 5422CBCL when larvae fed kernels not leaves of Bt corn. Additionally, *S. litura* presented a much stronger tolerance to 5422CBCL than 5422Bt1.

In 2015, genetically engineered (GE) corn expressing insecticidal crystalline (Cry) proteins derived from the bacterium *Bacillus thuringiensis* (Bt) were grown on more than 53.65 million hectares worldwide, covering 29% of global corn production area^1^. Bt corn hybrids are designed for protection against the most important corn pests, such as stem borers (e.g. European corn borer, *Ostrinia nubilalis*)^2^. However, a major threat to the long-term application of Bt corn is possible evolution of target pest resistance^3^,^4^. In addition, their efficiency for controlling some secondary lepidopteran pests has been questioned, raising concerns about potential outbreaks and their economic consequences^4^,^5^. González-Cabrera *et al*.^4^ have shown a much lower efficacy of Bt corn DKC6041YG (MON810, Cry1Ab) for controlling *Mythimna unipuncta* (Lepidoptera: Noctuidae)^4^. Other studies also have reported that *M. unipuncta* and *Helicoverpa armigera* could survive when they were fed Bt corn^6^,^7^. These secondary lepidopteran pests may display low susceptibility or develop resistance to Bt corn and gradually evolve into key pests, which has raised many concerns recently^8^,^9^,^10^.

There are multiple factors involved in the low susceptibility of secondary lepidopteran pests to Bt corn^7^,^11^. The first is sublethal or low levels of Cry toxin expressed in Bt corn, as well as a high rate of toxin elimination inside the insect gut^7^,^12^. The concentration of Cry toxins expressed in Bt corn hybrids varies in different tissues of plants throughout the growing season^8^,^13^,^14^. The highest level of Cry toxins is generally detected in heart leaves, and lower levels are found in pollen and kernels^8^,^14^. Cry1Ab levels of events Bt11 and MON810 ranged from 3 to 10 μgg^−1^ in fresh leaf and from 0.2 to 1.4 μgg^−1^ in kernels^15^. Moreover, Bt corn hybrids with different transformation events have different Cry1Ab levels. For example, Cry1Ab doses released from Bt11 Bt corn were higher than those of MON810 Bt corn^16^. Therefore, insects may encounter sublethal or low levels of Cry toxin when they were fed the respective tissues of Bt corn with lower levels of Cry toxin released, or Bt corn with transformation events having lower levels of Cry toxin expression. Additionally, insects can eliminate Cry toxins inside the gut through rapid excretion, or a high degradation rate inside this space, as occurs in *M. unipuncta* larvae^7^.

The second factor is the alteration of the mode of action of Cry toxins in the pest midgut^17^. The mode of action of Cry toxins has been well studied in lepidopteran insects, and it is widely accepted that the primary action of Cry toxins is to lyse midgut epithelial cells in the target pest by forming pores in the apical microvilli membrane of the cells^18^,^19^,^20^. The crystal inclusions (−130 kDa) ingested by susceptible larvae dissolve in the alkaline environment of the gut, and the solubilized inactive protoxins are cleaved by midgut proteases yielding 60–70 kDa protease resistant proteins^20^. The activated toxin then binds to specific receptors [e.g. cadherin-like receptors (CLR) and alkaline phosphatase (ALp)] located in the microvilli of the apical membrane of midgut epithelial cells. Oligomerization of the toxin leads to membrane insertion, pore formation, and cell lysis^20^,^21^,^22^. For lepidopteran insects, the proteolytic activation of protoxins and subsequent binding of the active toxin to receptors have been characterized as two crucial steps in mode of Cry1 action.

As Bt corn (Bt11 and Mon810) were transformed with truncated Cry1Ab gene corresponding to activated toxin, insect could ingest activated toxin without proteolytic activation of protoxin. Hence, binding of active toxin to receptors is a crucial step in the mechanism of Bt corn affecting insect. However, as activation and binding progress dynamically, it is likely that after the ingestion of the truncated toxin from transgenic corn, insects with low susceptibility could remove most of this toxin from the midgut before it can be fully activated to exert its toxic action^4^,^7^. Therefore, changes in the expression levels of receptors and the amounts of toxin injection in the midgut possibly results in insect low susceptibility to Bt corn^7^.

In addition to the direct toxic action of Cry toxins, a defense reaction may be induced at the same time in the insect midgut, including some enzymatic systems that provide resistance to Cry toxins: esterases, glutathione-S-transferases (GSH-ST), and cytochrome P450 monooxygenases^23^. It might be reasonable to assume that secondary lepidopteran pest sensitivity to Bt toxins can be significantly lowered by insect defense responses^24^. Therefore, insects low susceptibility to Bt corn should be the sum total of these above factors.

The common cutworm, *Spodoptera litura* Fabricius (Lepidoptera: Noctuidae), a serious crop pest with a strong migratory ability in the world, is widely distributed throughout Middle East, East Asia, Oceania, and Pacific islands, with the climate type ranging from tropical to temperate regions^25^,^26^. *S. litura* larvae are polyphagous with 389 host plants including >30 cultivated crops, such as cotton, corn, soybean, groundnut, vegetables, etc^27^,^28^. They move in large packets from one host plant to another, causing substantial economic loss to many field crops^29^,^30^. In the past decades, *S. litura* has developed high levels of resistance to insecticides^31^,^32^,^33^ and also low susceptibility to transgenic Bt cotton^34^. In the conventional corn field, we can find *S. litura* larvae damage corn by feeding on whorl leaves, corn bracts and fresh kernels. To investigate the possibility of *S. litura* low susceptibility to Bt corn, we conducted the following experiments: 1) an insect bioassay with *S. litura* larvae being fed artificial diet, to which different concentrations of Cry1Ab toxin incorporated into the diet; 2) an insect bioassay with *S. litura* larvae being fed different tissues (leaves and kernels) of two Bt corn hybrids [5422Bt1 (Event Bt11), 5422CBCL (MON810)] expressing Cry1Ab; 3) an insect bioassay was carried out with *S. litura* larvae by bagging on two Bt corn hybrids in greenhouse; 4) after larvae being fed different tissues (leaves or kernels) of two Bt corn hybrids, the detection of Cry1Ab, gene expression of Cry1A receptors and the enzyme activities were investigated.

## Results

### Effects of Cry1Ab toxin exposure on *S. litura* larvae

**Figure 1:**
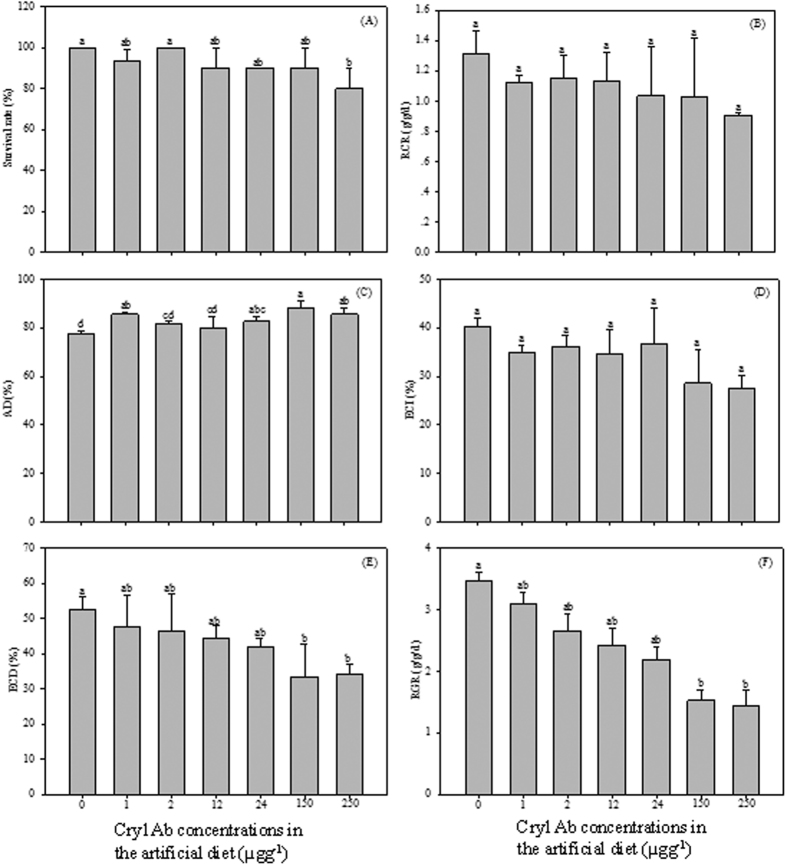
Effects of Cry1Ab toxin exposure on survival, growth and food utilization of *S. litura* larvae. After *S. litura* being fed Cry1Ab toxin contaminated artificial diet for 7 d, the survival rate of *S. litura* larvae (**A**), the relative consumption rate (RCR) (**B**), approximate digestibility (AD) (**C**), efficiency of conversion of ingested food (ECI) (**D**), efficiency of conversion of digested food (ECD) (**E**), relative growth rate (RGR) (**F**) of *S. litura*. Results are means ± SE. Bars marked with the same lowercase letter within a column were not significantly different.

After the 3^rd^ instar larvae were fed Cry1Ab toxin contaminated artificial diets for 7 d, no significant differences in the survival rate of *S. litura* were observed between control and treatments, except for the 250 μgg^−1^ treatment (Fig. 1A). More than 90% of total larvae could survive well in 0–150 μgg^−1^ treatments over a period of 7 d. However, the survival rate of larvae from 250 μgg^−1^ treatment was almost 80%.

The approximate digestibility (AD) and efficiency of conversion of digested food (ECD) of *S. litura* larvae compared to the control showed significant differences in the following Cry1Ab concentrations treatments, 150 and 250 μgg^−1^ (Fig. 1C,E). Significantly higher AD were observed in larvae from 1, 24, 150 and 250 μgg^−1^ Cry1Ab treatments, compared to control (Fig. 1C). After being fed 150 and 250 μgg^−1^ Cry1Ab supplemented diets, larvae ECD decreased significantly in comparison with control (Fig. 1E). No significant differences between control and treatments were observed in relative consumption rate (RCR) and efficiency of conversion of ingested food (ECI) of *S. litura* (Fig. 1B,D).

A decline was found in relative growth rates (RGR) of larvae provided Cry1Ab in the diets (Fig. 1F). RGRs of larvae from 150 and 250 μgg^−1^ Cry1Ab treatments were significantly lower than control. The RGR of larvae fed 250 μgg^−1^ Cry1Ab diets was 1.43 g/g/d, which was 46.36%, 54.07%, 59.18%, 65.77%, 94.24% and 41.55% of that of other treatments (1, 2, 12, 24, 150 μgg^−1^ treatment) and control, respectively.

### Effects of Bt corn tissues on *S. litura* larvae

**Figure 2:**
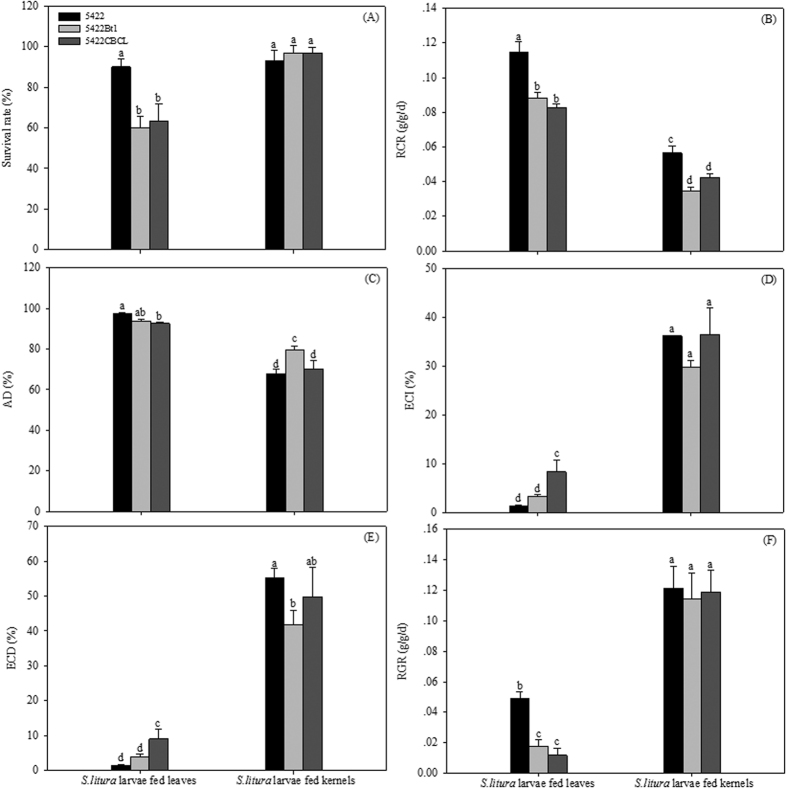
Effects of Bt corn different tissues on survival, growth and food utilization of *S. litura* larvae. After *S. litura* being fed leaves or kernels of three corn varieties for 7 d, the survival rate of *S. litura* larvae (**A**), relative consumption rate (RCR) (**B**), approximate digestibility (AD) (**C**), efficiency of conversion of ingested food (ECI) (**D**), efficiency of conversion of digested food (ECD) (**E**), the relative growth rate (RGR) (**F**) of *S. litura* were investigated. Results are means ± SE. Bars marked with the same lowercase letter within a column were not significantly different.

After *S. litura* larvae were fed the leaves of two Bt corn hybrids for 7 d, the survival rates of larvae were 60% and 63.33% for 5422Bt1 and 5422CBCL treatments, respectively, with a significant difference to that of the 5422 diet (90%). The survival rate of *S. litura* larvae fed kernels was higher than 93%, regardless of whether they received Bt or non-Bt corn (Fig. 2A).

After being fed the corn leaves, *S. litura* larvae in the control treatment showed significantly higher RCR and similar AD, ECI and ECD with those of 5422Bt1 treatment (Fig. 2B,C,D,E). The significant differences between 5422 and 5422 CBCL treatments were found in RCR, AD, ECI and ECD of larvae consuming leaves, where larvae from 5422 CBCL treatments showed significantly lower RCR, AD and significantly higher ECI and ECD (Fig. 2B,C,D,E). Under kernels treatments, larvae in the 5422Bt1 treatment presented significantly higher AD and lower RCR, ECI and ECD than that in control treatment; larvae fed 5422CBCL presented the similar AD, ECI and ECD, and significantly lower RCR in comparison with that of 5422 treatment (Fig. 2B,C,D,E).

After *S. litura* larvae were fed the leaves of 5422Bt1 and 5422CBCL, RGRs were 0.017 and 0.012 g/g/d, respectively, which were significantly lower than that of 5422 treatment (0.049 g/g/d). No significant differences between the three corn varieties were found in RGRs of *S. litura* consuming kernels (Fig. 2F).

### Effects of Bt corn on *S. litura* in greenhouse

**Figure 3:**
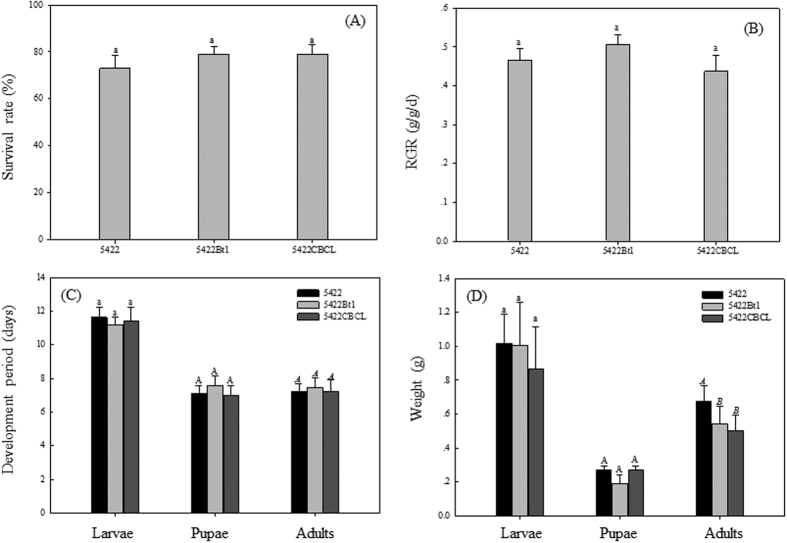
Effects of two Bt corn hybrids on *S. litura* in greenhouse. After *S. litura* being bagged on plants of three corn varieties, the survival rate (**A**), the relative growth rate (RGR) (**B**), development periods (**C**), and weights of *S. litura* larvae, pupae and adults (**D**) were investigated. Results are means ± SE. Bars marked with the same lowercase letter within a column were not significantly different; values labeled by the same small, capital or italic letters within a column were not significantly different.

After the 3^rd^ instar larvae were bagged on the corn plants, no significant differences among three corn varieties treatments were observed for the survival rate (Fig. 3A), RGR (Fig. 3B), development periods (Fig. 3C) and weight of larvae and pupae (Fig. 3D). Larvae bagged on 5422 had a significantly higher adult weight than those bagged on 5422Bt1 and 5422CBCL (Fig. 3D).

### Cry1Ab concentration in Bt corn and *S. litura*

We detected the Cry1Ab concentrations in the following samples by Cry1Ab/Ac enzyme-linked immunosorbent assay (ELISA), the diets, the gut contents, feces, midgut of larvae fed Cry1Ab toxin incorporated diets and the three corn varieties treatments.

**Figure 4:**
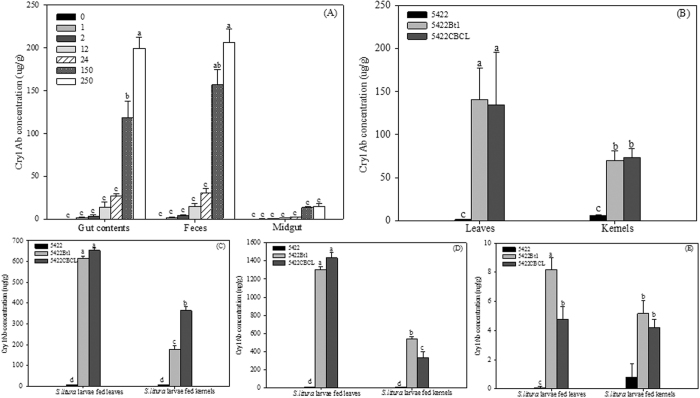
Cry1Ab concentrations in the corn and *S. litura* larvae. (**A**) Cry1Ab concentrations in the gut contents, feces and midgut of larvae fed Cry1Ab toxin contaminated artificial diet; (**B**) Cry1Ab concentrations in corn leaves and kernels; (**C**,**D**,**E**) Cry1Ab concentrations in gut contents, feces, midgut of larvae fed corn leaves and kernels; Results are means ± SE. Bars marked with the same lowercase letter within a column were not significantly different.

There were 1.373 ± 0.567 ngg^−1^, 2.021 ± 0.556 μgg^−1^, 3.328 ± 1.029 μgg^−1^, 11.494 ± 3.937 μgg^−1^, 33.596 ± 5.345 μgg^−1^, 149.431 ± 10.293 μgg^−1^, and 563.591 ± 36.598 μgg^−1^ Cry1Ab in the diets from 0, 1, 2, 12, 24, 150 and 250 μgg^−1^ Cry1Ab treatments, respectively. The Cry1Ab concentrations in the gut contents, feces and midguts increased with the increase of Cry1Ab toxin concentrations in artificial diets (Fig. 4A). Cry1Ab concentration in the gut contents of larvae fed 250 μgg^−1^ Cry1Ab toxin incorporated diets was 199.44 ± 13.33 μgg^−1^, which was 103.33, 54.28, 14.64, 7.32 and 1.69 times as that of other treatments (1, 2, 12, 24 and 150 μgg^−1^ Cry1Ab treatments), respectively. After larvae were fed 150 and 250 μgg^−1^ Cry1Ab toxin incorporated diets, Cry1Ab concentrations in the feces were significantly higher than that of other treatments. The Cry1Ab concentrations in the midgut of larvae fed 250 μgg^−1^ Cry1Ab toxin incorporated diets was 14.69 ± 3.33 μgg^−1^, which was 54.41, 32.64, 12.45, 6.20 and 1.06 times of that of other treatments (1, 2, 12, 24 and 150 μgg^−1^ Cry1Ab treatment), respectively. After larvae were fed 150 and 250 μgg^−1^ Cry1Ab toxin incorporated diets, the highest Cry1Ab concentration was detected in larval feces, which was significantly higher than that in larval midgut.

We also detected Cry1Ab concentrations in the following samples, leaves and kernels of the three corn varieties. No significant difference between 5422Bt1 and 5422CBCL was found in Cry1Ab concentrations in the leaves or kernels. Cry1Ab concentrations in the kernels of 5422Bt1 and 5422CBCL were 70.78 and 73.85 μgg^−1^, respectively, which were significantly lower than those in the leaves, being approximately half of those in corresponding leaves (Fig. 4B).

The obvious immunoreactive Cry1Ab was observed in the gut contents, feces of *S. litura* larvae from Bt corn treatments, regardless of corn tissues (Fig. 4C,D). The significantly higher Cry1Ab concentrations were detected in gut contents of larvae fed leaves than kernels, regardless of Bt corn hybrids. There were no significant differences between 5422Bt1 and 5422CBCL found in Cry1Ab concentrations of the gut contents of larvae fed leaves. When larvae fed 5422Bt1 kernels, Cry1Ab concentration in the gut contents was 177.78 ngg^−1^, which was 48.83% of 5422CBCL treatment. The significantly higher Cry1Ab concentrations were detected in the feces of larvae fed leaves than kernels, regardless of Bt corn hybrids (Fig. 4D). The Cry1Ab concentrations in the feces of larvae fed leaves of 5422Bt1 and 5422CBCL treatment were 1306.53 and 1433.51 ngg^−1^, which were 2.42 and 4.34 times that in larval feces of kernels treatments, respectively.

After being fed leaves or kernels of Bt corn for 7 d, the obvious immunoreactive Cry1Ab was observed in larval midgut (Fig. 4E). The Cry1Ab concentration in the midgut of larvae fed 5422Bt1 leaves was 8.19 ngg^−1^, which was 1.72 times that in the midgut from 5422CBCL treatment. The Cry1Ab concentration in the midgut of larvae fed kernels of 5422Bt1 was 5.16 ngg^−1^, which was higher than that of 5422CBCL treatment (4.18 ngg^−1^). The Cry1Ab concentration in the midgut of larvae fed 5422Bt1 leaves was significantly higher than that of kernels treatment.

After larvae being fed leaves, the highest Cry1Ab concentration was detected in larval feces, which was significantly higher than that in larval gut contents and midgut, regardless of 5422Bt1 and 5422CBCL. After larvae were fed 5422Bt1 kernels, the highest Cry1Ab concentration was detected in larval feces, which was significantly higher than that in larval gut contents and midgut. However, no significant difference between gut contents and feces was found in that of 5422CBCL kernel treatments.

### Cry1Ab immunoblot in corn tissues and *S. litura*

**Figure 5:**
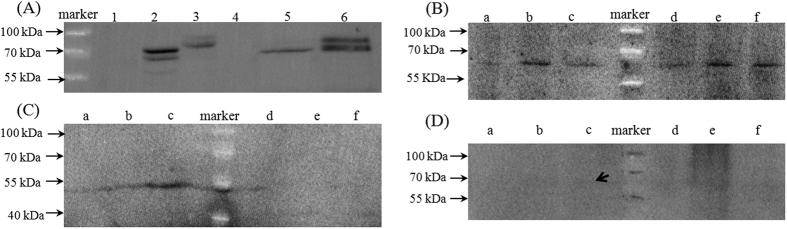
Western blot analysis of Cry1Ab fragments in corn tissues and *S. litura* larvae. (**A**) corn tissues, samples from leaves of 5422 (1), 5422Bt1 (2), 5422CBCL (3), kernels of 5422 (4), 5422Bt1 (5), 5422CBCL (6), respectively. (**B**) gut contents; (**C**) feces; (**D**) midgut, the small arrow in figure means a vague band; (**B**,**C**,**D**) samples from larvae fed leaves of 5422 (a), 5422Bt1 (b), 5422CBCL (c), kernels of 5422 (d), 5422Bt1 (e), 5422CBCL (f), respectively.

Western blot analysis revealed a considerable difference of Cry1Ab in the gut contents, feces and midgut of *S. litura* when they were fed different diets (leaves or kernels) (Fig. 5B,C,D). Cry1Ab extracted from leaves or kernels of 5422Bt1 and 5422CBCL had the molecular weight of approximately 72 and 90 kDa, respectively (Fig. 5A). In the gut contents of *S. litura* consuming Bt corn, a protein fragment of about 65 kDa was detected (Fig. 5B). In the feces of *S. litura* consuming leaves of Bt corn, only an protein fragment of about 50 kDa was detected (Fig. 5C), whereas almost no protein band appeared in feces of larvae fed Bt corn kernels (Fig. 5C). A weak band of about 65 kDa protein was detected in midgut of *S. litura* fed 5422CBCL leaves (Fig. 5D), whereas almost no fragments appeared in midgut of *S. litura* fed Bt kernels and 5422Bt1 leaves (Fig. 5D).

### Gene expression of Cry1Ab receptor in *S. litura* larvae midgut

**Figure 6:**
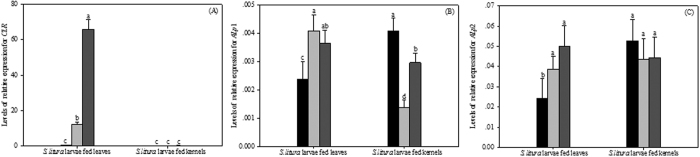
Effects of two Bt corn hybrids on Cry1Ab receptors expression in *S. litura* larvae midgut. (**A**) expression of cadherin-like receptors in midgut of *S. litura* fed corn leaves or kernels; (**B**,**C**) expression of *SlALp*1/2 in midgut of *S. litura* fed corn leaves or kernels. Results are means ± SE. Bars marked with the same lowercase letter within a column were not significantly different.

When *S. litura* larvae were fed 5422 leaves, the ratio of *SlCLR*/β-actin was 0.540, which was significantly lower than those of 5422Bt1 and 5422CBCL treatments, being 4.3% and 0.8% of that level, respectively (Fig. 6A). However, no significant differences among the three corn varieties were found in case of the kernels treatments.

Higher levels of *SlALp*1 were detected in *S. litura* larvae from Bt corn treatments, when they were fed leaves (Fig. 6B). When *S. litura* larvae were fed 5422 leaves, the ratio of *SlALp*1/β-actin was 0.0023, which was significantly lower than those of 5422Bt1 and 5422CBCL treatments, being 58.29% and 65.07%, respectively. After being fed the kernels, the highest level of *SlALp*1 was detected in larval midgut samples from 5422 treatment, being 2.98 and 1.37 times those of 5422Bt1 and 5422CBCL treatments, respectively.

The lowest level of *SlALp*2 expression was detected in the midgut from 5422 treatment, and showed a significant difference from that of 5422Bt1 treatment, when they were fed leaves (Fig. 6C). After being fed Bt corn leaves, the ratio of *SlALp*2/β-actin in larval midgut from 5422CBCL treatments was about 0.050, which was significantly more than that of 5422 treatments. Although *SlALp*2 expression levels in larvae fed 5422CBCL kernels was lower than that of 5422 treatment, no significant difference was found between the two.

### Enzyme activities of *S. litura* larvae midgut

**Figure 7:**
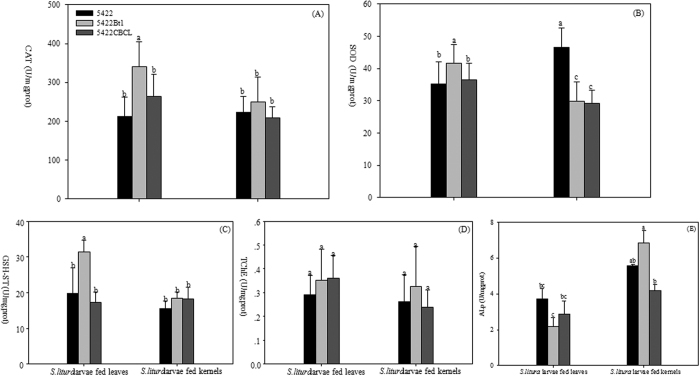
Effects of two Bt corn hybrids on enzyme activities of *S. litura* larvae midgut. After *S. litura* larvae being fed leaves or kernels of three corn varieties for 7 d, the following midgut enzyme activities were determined: catalase (CAT) (**A**), superoxide dismutase (SOD) (**B**), glutathione-S-transferase (GSH-ST) (**C**), true choline esterase (TChE) (**D**), alkaline phosphatase (ALp) (**E**). Results are means ± SE. Bars marked with the same lowercase letter within a column were not significantly different.

When *S. litura* larvae were fed the 5422Bt1 leaves, the enzymes, including catalase (CAT), superoxide dismutase (SOD), and GSH-ST, detected in this experiment showed significantly higher activities than those of the 5422 and 5422CBCL treatments (Fig. 7). After being fed the 5422CBCL leaves, no significant differences between 5422 and 5422CBCL treatments were observed in activities of all enzymes. No significant difference among three corn varieties was present in CAT, GSH-ST and true choline esterase (TChE) when *S. litura* larvae fed kernels (Fig. 7A,C,D). When *S. litura* larvae were fed the Bt corn kernels, SOD activities were significantly lower than those of 5422 treatments (Fig. 7B). Compared different diets treatments from 5422Bt1, CAT, SOD, and GSH-ST activities in the midgut from leaf treatments were significantly higher than those of kernel treatments. No significant difference in treatments between leaves and kernels was present in the detected enzymes of larvae fed 5422CBCL, except SOD. When *S. litura* larvae were fed the corn leaves, no significant difference among three corn varieties treatments was found in the ALp activity in larval midgut (Fig. 7E). After being exposed to the kernels, ALp activity in midgut from 5422Bt1 treatments was significantly higher than those of 5422 CBCL treatments, while no significant difference was found between 5422 and 5422CBCL treatments. Compared different diets treatments from 5422Bt1, ALp activity in larval midgut from leaf treatments were significantly lower than that of kernel treatments. No significant difference in treatments between leaves and kernels was present in the ALp activity of larvae fed 5422CBCL and 5422.

## Discussion

Bioassays carried out to determine the effects of Cry1Ab-contaminated diets on *S. litura* indicated that larvae exhibited the expected response, where the negative effect of Cry1Ab ingestion on the survival and growth increased with increasing Cry1Ab toxin concentration in diet. This result was consistent with other studies, in which insects fed diets with Cry toxins typically displayed reduced growth, food consumption and utilization efficiency^12^,^35^,^36^,^37^. Although Cry1Ab concentrations in these diets (563.59 ± 36.60 μgg^−1^ in diets from 250 μgg^−1^ Cry1Ab treatments) were 4–8 times as high as in leaves and kernels of 5422Bt1 and 5422CBCL, which can be seen as the ‘worst-case scenario’ in the lab tests, the acute lethal effects of Cry1Ab toxin on *S. litura* larvae were not present, with high survival rates (80%) for treatments with the highest Cry1Ab toxin concentration. Bioassays carried out to determine the effects of Bt corn tissues on *S. litura* performance indicated that *S. litura* larvae could survive well and complete their development in 5422Bt1 and 5422CBCL if they were fed kernels of these varieties instead of leaves. *S. litura* larvae fed the kernels exhibited similar metabolic expenditures when fed either Bt or non-Bt corn, as reflected on ECI and ECD values. Moreover, AD of *S. litura* larvae fed with 5422Bt1 kernels was significantly higher than when fed with 5422 kernels, indicating that nutrient utilization was not impaired. These findings highlight the low susceptibility of *S. litura* to tested two Bt corn hybrids. Furthermore, the results from greenhouse test with *S. litura* bagging on Bt corn plants indicated that there were no detrimental effects on larval survival, RGR, development time, and weight of larvae, pupae or adults.

In view of three bioassays together, *S. litura* larvae showed low susceptibility to tested two Bt corn hybrids. González-Cabrera *et al*.^4^ also described that available Bt corn cultivars show a much lower efficacy for controlling some secondary Spanish corn lepidopteran pests, such as the true armyworm, *M. unipuncta*^4^. Pérez-Hedo *et al*.^7^ demonstrated that the Cry1Ab concentration expressed in transgenic corn hybrids represents a ‘low dose’ scenario for *M. unipuncta* and *H. armigera*^7^. Additionally, Erasmus *et al*.^38^ indicated that Bt corns have not any significant effect on the control of *Agrotis segetum* under field conditions, as feeding on Bt corn did not affect survival of 1^st^ or 4^th^-instar larvae^38^.

Some reports have indicated that larger lepidopteran larvae are more Bt tolerant than smaller larvae^12^,^39^,^40^,^41^. Bt corn (MON810) significantly reduced survival, developmental rate and pupal and adult weight of *Busseola fusca* compared with parental line. These differences were more pronounced with 2^nd^ instar larvae than with 3^rd^ instar larvae^41^. Valadez-lira *et al*.^39^ found that 2^nd^ instar lepidopteran larvae were susceptible to Bt toxin and the effective degrees depend on different insect species, whereas Bt toxin was not effective on 4^th^ instar larvae^39^. However, as same as other developing stages, 1^st^ instar larvae of *S. litura* were not susceptible to GK-12 (Bt cotton expressing Cry1Ab/Ac) compared with control^30^,^42^. The effects of Bt corn on life history parameters of *Chilo partellus* were similar for tested 4- and 17- day-old larvae^37^. Erasmus *et al*.^38^ showed that Bt corn did not affect the survival of 1^st^ instar larvae of *A. segetum*^38^. Likewise, Bt corn (HD-1) was not effective on 1^st^ instar larvae of *Agrotis ipsilon* and *Papaipema nebris*^5^. Additional reports indicated Bt corn had no effects on the 3^rd^ instar or older lepidopteran larvae^5^,^7^,^38^. Therefore, it was inferred that the effects of Bt toxin or Bt plants on lepidopteran larvae varying with the age depend on insect species. The caterpillars of genus *Spodoptera*, such as *S. litura*, are not fully susceptible to the current Bt cotton^30^,^42^. In this study, we conducted three bioassays with the 3^rd^ instar larvae of *S. litura* and the results suggested that they were low susceptibility to tested two Bt corn hybrids if they were fed kernels of these varieties instead of leaves. As polyphagous pest, *S. litura* larvae feed 389 host plants and move in large packets from one host plant to another, so it is easy for neonates to avoid poor quality diets^26^,^27^. Furthermore, *S. litura* larvae are known for cutting damage to plants and can cause the highest yield losses to plants when 3^rd^ through 6^th^ instars feed on plants^27^, which is more realistic to rear larvae on diets until the 3^rd^ instar before using them in experiments.

Van Rensburg (2001) reported that susceptibility of *B. fusca* larvae to Bt corn plants differed significantly, depending on what part of the plant was being attacked^43^, which was similar with our results. Additionally, several of factors for the low susceptibility of insect species to Cry toxins were also revealed in this study. The first was avoidance of feeding the tissues with high Cry1Ab content. Cry1Ab levels present in 5422Bt1 and 5422CBCL were significantly higher in leaves than in corresponding kernels (Fig. 4B), which was consistent with that Cry1Ab amounts in leaves were more than that in kernels of Bt corn hybrids (Bt11 and MON810)^7^. The Cry1Ab level in Bt crops varies between tissues^8^,^13^,^14^,^16^, which affects the quality of crops as food sources of target or secondary pests^44^,^45^,^46^, and presumably results in the decrease of pest susceptibility to Bt crops^8^,^47^. For example, the mortality of *Ostrinia nubilalis* fed kernels of MON810 Bt corn was significantly lower than those fed leaves, which was due to the difference in Cry1Ab expression in kernels and leaves^7^. Furthermore, MacIntosh *et al*.^48^ established that the LC_50_ value of Cry1Ab protein for European corn borer was about 9 times lower than for corn earworm, and 24 times lower than for black cutworm^48^. It might be reasonable to assume that the Cry1Ab expression tested in two Bt corn hybrids presented “low dose” scenario to the cutworm *S. litura*.

In addition to the ‘low dose’ scenario of Bt corn for *S. litura*, toxin ingestion, degradation and excretion that was investigated in *S. litura* larvae could be another factor to explain the lower Cry1Ab levels in the midgut. In the present study, *S. litura* ingested more and more Cry1Ab (Cry1Ab in the gut contents, Fig. 4A) from food with the increase of Cry1Ab concentrations in diets; and then, they remove most of Cry1Ab toxin from the midgut via feces and only a small fraction was absorbed by midgut (Under 250 μgg^−1^ Cry1Ab toxin treatment, Cry1Ab concentration in the feces was 14.06 times of that in larval midgut).

Likewise, *S. litura* larvae ingested Cry1Ab from Bt plants, and significant Cry1Ab amounts could be excreted via feces, only a small part was absorbed by midgut (Cry1Ab concentration in feces was almost 100 times of that in midgut, Fig. 4D,E). The significantly higher Cry1Ab contents in the gut contents and significantly higher AD were found in larvae fed leaves than kernels treatment (Figs 4C and 2C), which suggested that food is retained longer in the midgut which may increase the interactions between the enzymes and the Cry1Ab present in the midgut^49^. Although Cry1Ab concentrations in the feces of leaf treatments were significantly higher than kernels treatments, the difference in AD reflected that *S. litura* larvae fed Bt corn leaves could ingest more food and reduce feces production. This may indicate a significant reduction in Cry1Ab excretion in leaf treatments, compared to kernel treatments. Janmaat *et al*.^12^ also showed that susceptible larvae exhibited a reduction in feces production at all tested Bt concentrations^12^.

Additionally, the different molecular weights of Cry1Ab protein in plants (72 kDa and 90 kDa for 5422Bt1 and 5422CBCL, respectively), gut contents (65 kDa) and feces (50 kDa) showed that after ingested Cry1Ab protein from plants, *S. litura* may degrade Cry1Ab protein into small fragment during digestion through the gut. No protein fragments were blotted in the feces material from kernels treatments although the amounts of Cry1Ab were higher or similar with that in the gut contents detected by DAS-ELISA, which further suggested that *S. litura* fed kernels could ingest less Cry1Ab and digest more Cry1Ab protein into little fragments with a non-active form. Lutz *et al*.^50^ showed that the antibody used in the ELISA kit employed in our study reacts also with degraded fragments of the Cry1Ab protein of approximately 17 and 34 kDa sizes^50^. Thus it seems likely that fragments smaller molecular weight could be quantified with the immuno-assay but then were too small for our Western Blot technique. This corresponds to results from Emmerling *et al*.^51^, who also found fragments smaller than 17 kDa size were quantified by DAS-ELISA which in turn could not be detected in the Western Blot^51^.

The amount of Cry1Ab was further significantly decreased between gut contents and midgut of the cutworms. Cry1Ab concentration was nearly completely degraded in kernel treatments, from 177.78 and 364.02 ng g^−1^ in the gut contents to 5.16 and 4.28 ng g^−1^ in the midgut for 5422Bt1 and 5422CBCL treatments, respectively. Western blot also showed no protein fragments in the midgut of kernels treatments, whereas a weak protein band of about 65 kDa (it was consistent with protein fragment in the gut contents) was found in the midgut of 5422 CBCL treatments (Fig. 5E).

This may explain lower Cry1Ab levels in the insect midgut, as Rees *et al*.^52^ indicated for a number of lepidopteran larvae^51^. Besides, higher Cry1Ab amounts were stored in the midgut of *S. litura* larvae reared on leaves compared with kernels treatment, regardless of whether 5422Bt1 or 5422CBCL were supplied (Fig. 4E). For different diets, the difference in the Cry1Ab amounts in midgut of *S. litura* larvae was consistent with differences in their growth and food utilization.

Importantly, remarkable differences of gene expression in Cry1Ab reporters (CLR and ALp) were found between leaves and kernels of Bt corn hybrids. The expression levels of two Cry1Ab receptors from larvae fed Bt corn leaves were significantly higher than those of 5422 treatments, whereas no significant difference was found in the kernel diets among three corn varieties (Fig. 6). As activation and binding progress dynamically, it was inferred that larvae fed Bt corn kernels presented significantly lower binding activated Cry1Ab to specific receptors in midgut than larvae from leaf treatment, which may partially account for its low susceptibility to tested two Bt corn hybrids.

Generally, there is energy resources trade-off among growth, detoxification and immune responses of organisms^52^. Since high AD and RCR, and low ECI, ECD and RGR were found in leaf diets in contrast to kernels treatments (Fig. 2), *S. litura* larvae apparently consumed more food while not gaining weight, which suggested a diversion of energy from biomass production to detoxification. Under sublethal conditions, the insects also adaptively responded to changes in their environment by altering detoxification and immune system responses, including changes in activities of protective and detoxifying enzymes as well as others^53^. In this work, we showed a significant increase in the activity of CAT, SOD, and GSH-ST from 5422Bt1 leaf diet compared to that of 5422 (Fig. 7), suggesting that *S. litura* larvae were experiencing negative environmental factors, such as Cry1Ab expression in 5422Bt1 leaves and larvae midgut. However, no significant differences of enzyme activity were observed between 5422CBCL and 5422 diets.

Interestingly, when *S. litura* larvae were fed the corn leaves, no significant difference among three corn varieties was found in the ALp activity of larval midgut. This pattern was inconsistent with *SlALp* gene expression level, where the *SlALp*1/2 expression levels from Bt corn leaf treatments were significantly higher in *S. litura* larvae fed non-Bt corn. The possible reason was that gene expression was not positively correlated with protein synthesis. Messenger RNAs are rapidly changed by external or internal stimuli and thereby alter the composition of the transcriptome within hours^53^. After being exposed to the 5422Bt1 kernels, ALp activity in larval midgut was significantly higher than those of 5422 treatments, while no significant difference was found between 5422 and 5422CBCL treatments. The contrast pattern was present in *SlALp*1 gene expression from 5422Bt1 kernels treatment. Yan *et al*.^55^ showed that role of ALp in the insecticidal mechanism of Bt toxin may involve in two aspects: 1) ALp binds Bt toxin in brush border membrane vesicles (BBMV) of midgut as a receptor; 2) the reduce of ALp activity in migut resulted in an imbalance of physiological metabolism^54^. Therefore, when *S. litura* larvae were treated with Bt corn expressing Cry1Ab, the contrary pattern may exist in ALp activity and gene expression. This was consistent with the findings described by several reports. Arenas *et al*.^56^ found that the higher levels of ALp genes were detected in gut of *Manduca sexta* larvae that were more susceptible to Bt toxin^55^, and it was the same case in *Helicoverpa armigera* larvae^56^,^57^. Jurant-Fuentes and Adang (2004, 2006) reported that the down-regulation of ALp in *Helicoverpa virescens* larvae that were resistance to Cry1Ac^58^,^59^. Nevertheless, the specific binding between Bt toxin and ALps located in BBMV of midgut under native conditions resulted in inhibition of ALp activity in susceptible pests including *M. sexta, H. virescens* and *Aedes aegypti*^58^,^60^,^61^,^62^,^63^. Therefore, the inconsistent pattern was found in ALp activity and *SlALp*1/2 genes expression levels, when *S. litura* larvae were fed leaves or kernels of two Bt corn hybrids, which was associated with the role of ALp in the insecticidal mechanism of Bt toxin.

When chronic effects of two Bt corn hybrids leaves were compared, we found that 5422CBCL (MON810) was not more serious to *S. litura* to a certain extent. Here, larvae from 5422CBCL treatments showed significantly higher ECI, ECD and lower AD than that of 5422Bt1 treatments (Fig. 2C,D,E), suggesting that they reduced food eaten and increased feces production, and finally might exhibit reducing Cry1Ab levels in larval gut of 5422CBCL treatments. This predicted case was consistent with our results of Cry1Ab levels detected in the midgut (Fig. 4E). Furthermore, the differences in *S. litura* defense response present between 5422Bt1 and 5422CBCL treatments were also in agreement with above case. Therefore, *S. litura* showed a significantly lower susceptibility to 5422CBCL in comparison with 5422Bt1, which was consistent with what described by Saxena *et al*.^16^, that is larvae fed Bt corn with MON810 displayed lower susceptibility than those fed Bt11^16^. According to Saxena *et al*.^16^, five Bt corn hybrids representing transformation Event Bt11, grown in the field presented stronger toxic activity to lepidopteran pest *M. sexta*, where the mean percentage mortality of *M. sexta* was 64.3%, and the mean of larval weight was 71 mg. However, six Bt corn hybrids representing transformation Event MON810 had lower mortality (53.7%) and higher larval weight (75.83 mg)^16^.

We demonstrated that *S. litura* have a low susceptibility to tested two Bt hybrids [5422Bt1 (event Bt11) and 5422CBCL (Mon810)] possibly by feeding tissues (kernels) expressing low Cry1Ab levels, by a high Cry1Ab degradation through gut, and by a high Cry1Ab excretion via feces. Their low susceptibility was reflected in the following aspects: 1) larvae displayed similar survival rates, growth and food utilization when feeding on kernels, but not leaves of two Bt corn hybrids and non-Bt corn (5422); 2) lower levels of *SlCLR, SlALp* genes were found in larvae fed kernels than leaves of both Bt corn hybrids; 3) the changes in the defense response to 5422Bt1, where the activities of CAT, SOD and GSH-ST in the midgut extracted from larvae fed 5422Bt1 leaves were significantly higher than those of 5422 leaves treatments. Additionally, *S. litura* larvae presented a much stronger tolerance to 5422CBCL (MON810) in comparison with 5422Bt1 (Bt11).

## Methods

### Corn plants

Two transgenic corn hybrids [5422Bt1 (event Bt11) and 5422CBCL (MON810) from Beck’s Hybrids, Atlanta, Indiana, USA] expressing Cry1Ab and their near-isoline cultivar (5422) were cultivated in a greenhouse (25 ± 1 °C, 75 ± 5% humidity, 16:8 h L: D regime). Plants were cultivated at distances of 40 cm, with each hybrid in a 3.0 m × 3.4 m plot (a total of three plots). During the maturation stage of corn, the fresh leaves and kernels were used to feed the insects.

### Insect culture

Eggs of *S. litura* were provided by the Insectarium of Institute of Tropical and Subtropical Ecology, South China Agricultural University. Upon hatching, the larvae were reared on the artificial diet^53^ under constant conditions of 27 °C, 65% relative humidity, and a 12 h light: 12 h dark photoperiod in a climatic cabin (MLR-350H, Sanyo Electric Biomedical Co., Ltd, Japan) until the experiments were carried out.

### Insect bioassay with Cry1Ab toxin

The insect bioassay was carried out with the 3^rd^ instar larvae of *S. litura* by feeding them an artificial diet^53^, to which different amounts of Cry1Ab toxin (Shanghai Youlong Biotech Co., Ltd., Shanghai, China) had incorporated individually. The final concentrations of Cry1Ab in diets were 0 (control), 1, 2, 12, 24, 150 and 250 μgg^−1^.

Approximately 200 eggs were put in sterilized Petri dishes (12 cm diameter). Upon hatching, fresh diets of the different treatments were introduced daily to the Petri dishes. When larvae reached the 3^rd^ instar, 30 larvae with similar size and weight (45–55 mg) from every treatment were chosen and transferred to plastic boxes (4 cm diameter) (Guangzhou Jianxin plastic products Co., Ltd., Guangzhou, China) to be reared individually with enough food for 7 d; each bioassay was performed in triplicate. The number of surviving larvae was recorded daily. They were individually weighed daily using an electronic balance (CPA224S, Sartorius AG, Goettingen, Germany). Larval survival rate was calculated as *N*_*n*_/*N*_*0*_ × 100, where *N*_*0*_is the number of *S. litura* larvae at the beginning of the experiment, and *N*_*n*_ is the number of larvae on day *n* of the experiment.

An understanding of the processes involved in larval nutrition of Lepidoptera was achieved with the nutritional indices defined by Waldbauer^64^. Nutritional indices, expressed as dry weight, were calculated as follows: RCR (g/g/d) = dry weight of food eaten/(duration of feeding (d) × mean dry weight of larvae during the feeding period); AD (%) = 100 × (dry weight of food eaten − dry weight of feces produced)/dry weight of food eaten; ECI (%) = 100 × (dry weight gain of larva/dry weight of food eaten); ECD (%) = 100 × dry weight gain of larva/(dry weight of food eaten − dry weight of feces produced); RGR (g/g/d) = dry weight gain of larvae during period/(duration of feeding (d) × mean dry weight of larvae during the period). The weight gain and the mean larval body weight were calculated using the following formulas: weight gain = final weight − initial weight; mean weight = (initial weight + final weight)/2.

### Insect bioassays using different tissues of Bt corn

*S. litura* 3^rd^ instar larvae with an average weight of about 50 mg (45–55 mg) were chosen and reared individually in plastic boxes (4 cm diameter) for 7 d. Diets (1.8–2 g) of fresh leaves or kernels were introduced daily into the plastic boxes, with 30 replicates per diet for each corn variety. The survival rate and food utilization of *S. litura* were recorded as designed in above section ‘Insect bioassay with Cry1Ab toxin’.

### Insect bioassay by bagging with Bt corn

The bioassay was conducted in greenhouse. The 7^th^ and 8^th^ leaf of each plant were enclosed in a mesh bag, and the 3^rd^ instar larvae were placed in bags (5 larvae per bag), 20 bags were prepared for each corn variety. Every 3 d, the bag was taken down, and the survival rate and average weight of all larvae were recorded. When larvae developed into pre-pupae, the bags were taken back to the lab. The survival rate of larvae as well as the RGR was determined. Furthermore, the weight of pupae and adults was also recorded.

### ELISA of Cry1Ab

The experimental design was as described in above section ‘Insect bioassays using different tissues of Bt corn’ and ‘insect bioassay with Cry1Ab toxin’. 2.0 g fresh weight of test leaves or kernels, and 2.0 g of the feces of *S. litura* larvae fed corn tissues (leaves or kernels) and Cry1Ab contaminated artificial diets were sampled, flash frozen, weighed, lyophilized and weighed again. Additionally, the tested larvae fed Cry1Ab contaminated artificial diets and corn tissues (leaves or kernels) were dissected in phosphate-buffered saline (PBS; 137 mM NaCl; 2.7 mM KCl; 8 mM Na_2_HPO_4_; 1.5 mM KH_2_PO_4_; pH 7.5), and the gut contents and midgut were collected. The samples were weighed and frozen immediately in liquid nitrogen and stored at −80 °C until being used for Cry1Ab detection.

The Cry1Ab concentrations in above samples were measured using a Cry1Ab/Ac enzyme-linked immunosorbent assay (ELISA) kits, following the manufacturer’s protocol [Catalogue number: PSP 06200 (Zhongjian Baotai Biological Technology Co., Ltd. Beijing, China); Agdia, Elkhart, Indiana, USA]. Absorbance was measured at 650 nm with a microplate reader (Molecular Devices, California, USA). The Cry1Ab concentration was calculated using a six-point standard curve developed with purified Cry1Ab (supplied with the kit). Test results were validated with both positive and negative controls.

### Immunoblot of Cry1Ab

The experimental design was as described in section ‘Insect bioassay with different tissues of Bt corn’. The collection of samples was as described in section ‘ELISA of Cry1Ab’. The concentration of total proteins extracted from the samples, including 2.0 g fresh weight of leaves or kernels on which *S. litura* had fed, and the gut contents, feces and midgut of 10 of the 6^th^ instar larvae fed corn leaves or kernels, were determined using the Bradford protein assay before immunoblotting. 5 μl protein marker (PageRulerTM Prestained Protein Ladder, ThermoFisher, Republic of Lithuania) and equal amounts of total protein (15 mg) extracted from above samples were separated by 10% sodium dodecyl sulphate polyacrylamide gel electrophoresis (SDS-PAGE) and transferred to nitrocellulose membranes (Optitran BA-S83, Schleicher and Schuell, Keene, NH, USA) in buffer (25 mM Tris, pH 8.3; 192 mM Glycine; 10% Methanol) using the Trans-Blot^®^ SD Semi-Dry Transfer Cell (Bio-Rad, Hercules, CA, USA) at 12 V for 40 min. The membrane was blocked with 5% fat-free milk for 2 h, and then incubated for 2 h with rabbit anti-Cry1Ab (Abcam, University of Cambridge, UK) diluted 1:500 in PBST. The membrane was washed three times with PBST for 15 min each wash, and then incubated for 1 h with goat anti-rabbit immunoglobulin G-horseradish peroxidase (IgG-HRP) (Sigma, St Louis, MO, USA) diluted 1:2000 in PBST. The membrane was washed again with PBST three times (15 min each wash) and the color was developed with the 3,3′,5, 5′-tetramethylbenzidine (TMB).

### Gene expression of Cry1Ab receptors in *S. litura*

The insect treatment was as described in section ‘Insect bioassay with different tissues of Bt corn’. Five surviving larvae were picked from every diet and washed in distilled water, with 3 replicates per treatment. After dissection, midgut samples were collected and frozen immediately in liquid nitrogen and stored at −80 °C until RNA extraction for each different treatment. Relative Quantitative PCR was used to investigate expression of CLR and two ALp genes in *S. litura*. Gene specific primers for QPCR were designed for *S. litura* cadherin-like receptor (*SlCLR*) (GenBank Accession no. JN687590) and two *S. litura* ALp cDNAs (GenBank Accession no. JN687588 (*SlALp*1); JN687589 (*SlALp*2)): QCLRS (5′-TGA TTA TGA CGA AGC AAT GAT GA-3′) and QCLRR (5′-GTG TAA CGG ACT CGG TTG TAG AG-3′), QALp1S (5′-ACT GAC TGC GAG GCC TCG GTG G-3′) and QALp1R (5′-GGC TTT CTT AAC GTC TGC ATC G-3′), QALp2S (5′-CGA GGC TGC GAG GAG TCC ACC-3′) and QALp2R (5′-ATG GTC CTC CAC CGC CTT GTT G-3′). Primers of the housekeeping gene β-actin were used as endogenous controls: QActinS (5′-TGA GAC CTT CAA CTC CCC CG-3′) and QActinR (5′-GCG ACC AGC CAA GTC CAG AC-3′). QPCR was performed on a DNA Engine Opticon 2 Continuous Fluorescence Detection System (MJ Research Inc., Waltham, MA, USA) with SYBR Premix Ex Taq Kit (Takara, Japan) under the following thermal program: one cycle of 95 °C for 10 s, 40 cycles of 95 °C for 5 s and 60 °C for 30 s. The method for the preparation of standard curve was adapted for Shu *et al*.^65^. The number of gene copies from unknown samples per reaction mix was determined by the appropriate standard curve based on the cycle number at the set threshold fluorescent intensity. The homogeneity of the PCR product was confirmed by melting curve analysis following PCR. The *SlCLR* and *SlAlp*1/2 mRNA levels were normalized to β-actin and results were expressed as a relative level (number of copies of target mRNA/number of copies of β-actin). Three replicates were performed for each reaction to account for intra experiment variation.

### Measurement of enzyme activities

The experimental design was as described in section ‘Insect bioassay with different tissues of Bt corn’. The collection of samples was as described in section ‘ELISA of Cry1Ab’. The following enzymes activities were detected: protective enzymes (CAT and SOD), detoxifying enzymes (TChE and GSH-ST), and ALp. After dissection, midguts were washed in 4 °C physiological saline (0.7% NaCl) and then dried on filter paper and weighed. The samples were homogenized in 9 times volume of cooled homogenate (0.01 M Tris-HCl, 0.0001 M EDTA-2Na, 0.01 M 0.8% NaCl with sucrose). After centrifugation at 2000* × g* and 4 °C for 15 min, aliquots of the supernatants were directly used for measurement of enzyme activities using the corresponding enzyme kit obtained from the Nanjing Jiancheng Biological Engineering Institute (NJBI, Nanjing, China).

### Data analysis

All statistical analyses were conducted using the software package SPSS (version 13; SPSS, Inc., Chicago, USA). For all tests, a significant level of 5% was applied. One-way ANOVA followed by Tukey HSD test was carried out to compare the differences among the treatments (including control) for the following parameters: survival rate of larvae, RGR, RCR, AD, ECI and ECD, Cry1Ab content, enzyme activities and gene expressions. The percentages of survival and food utilization were arcsine square-root transformed before analysis. Other data were transformed via the natural logarithm when necessary to verify variance homogeneity.

## Additional Information

**How to cite this article**: Yinghua, S. *et al*. Responses of the cutworm *Spodoptera litura* (Lepidoptera: Noctuidae) to two Bt corn hybrids expressing Cry1Ab. *Sci. Rep.* **7**, 41577; doi: 10.1038/srep41577 (2017).

**Publisher's note:** Springer Nature remains neutral with regard to jurisdictional claims in published maps and institutional affiliations.
